# Sequence and structural similarities of ACCase protein of *Phalaris minor* and wheat: An insight to explain herbicide selectivity

**DOI:** 10.3389/fpls.2022.1056474

**Published:** 2023-01-04

**Authors:** Bikash Kumar Rajak, Priyanka Rani, Nitesh Singh, Durg Vijay Singh

**Affiliations:** ^1^ Molecular Modelling and Computer Aided Drug Discovery Laboratory, Department of Bioinformatics, School of Earth, Biological and Environmental Sciences, Central University of South Bihar Gaya, Gaya, Bihar, India; ^2^ Department of Biosciences, University institute of Biotechnology, Chandigarh University, Mohali, Punjab, India

**Keywords:** *Phalaris minor*, *Triticum aestivum*, herbicide resistant, Acetyl-CoA Carboxylase, molecular modeling, molecular dynamics simulation, mutation, sequence and structure similarity

## Abstract

Uncontrolled growth of *Phalaris minor* in the wheat (*Triticum aestivum*) crop has remained a problem, leading to a massive reduction in wheat grain production. Herbicides have been used to control the weed, which leads to the development of frequent resistance in *P. minor* and mutant biotypes were also reported (Trp2027Cys and Ile2041Asn). Development of resistance enforced agro researchers to analyses the action of herbicide on *P. minor*. In this study, the sequence and structure of *P. minor* and *T. aestivum* Acetyl CoA Carboxylase (ACCase) have been analysed to locate the differences in their sequence and structure and to formulate a plausible explanation of the selectivity of herbicides which may help in the rationale discovery of noble herbicides. The sequence and 3D structure analysis of weed and wheat ACCase indicate minute differences in the distantly located amino acid residues. However, proteins are conserved at the binding site of herbicides with no mutation at the catalytic site. Analysis indicates that herbicides selectively target *P. minor* ACCase might be due to unknown other reasons, but not due to differences in their protein sequence and structure.

## 1 Introduction

Infestation of *Phalaris minor* (200* P. minor* plants/m^2^) has been a longstanding management problem that reduces the wheat yield by 25-50% (Franke et al., 2003; Bhan et al., 2006; Bhullar et al., 2016). A combination of agroecological factors provides a suitable environment for the growth and survival of weeds in wheat crop fields. Besides being morphologically similar to each other in their initial stage (plantlets), *P. minor* and *Triticum aestivum* also have the same optimal growth conditions (Temperature: 10-20°C and Humidity: 45-50%) (Bhan and Choudary, 1976). Under extreme cases, untreated weed infestation can result in total crop failure (Malik et al., 1981; Singh, 2007). Until the early 1990s, farmers used isoproturon (substitute for urea-based herbicide) a PSII inhibiting herbicide, which was first recommended in the north-western part of India in 1977-78 to control *P. minor* in the crop field. However, after 15 years of use, the first case of herbicide resistance in *P. minor* was confirmed in India in 1991 (Malik et al., 1981; Chhokar and Malik, 2002).

To overcome the herbicide resistance several new herbicides with different targets such as Acetyl-CoA Carboxylase (ACCase) and Acetolactate synthase (ALS) were introduced in the market. Multiple and cross-resistance against the newly introduced alternate herbicides were soon reported in India (Chhokar et al., 2012; Kaur et al., 2017), South Africa (Pieterse, 2010) as well as in Israel regions against Aryloxyphenoxypropionate *aka* FOP (fenoxaprop and diclofop) group of herbicides (Gherekhloo et al., 2011; Gherekhloo et al., 2012). To control the resistant biotypes of *P. minor* 2-8 times higher doses of isoproturon herbicide were required as reported by Malik and Singh in 1995. The report of resistance against the newly introduced herbicides was recognised and it has been determined that herbicides already on the market are not enough. There is a need to develop new potential herbicides to control the problem of *P. minor* infestation.

Acetyl-CoA Carboxylase (ACCase) is considered a proficient target for the development of herbicides against weeds of the Poaceae family. There are two distinct isoforms of ACCase: the plastid ACCase (heteromeric) and the cytosolic ACCase (homomeric) (Yu et al., 2007). Heteromeric ACCase i.e., plastid ACCase comprises four independent polypeptide units: Biotin Carboxylase- BC, Biotin Carboxyl Carrier protein- BCCP and two carboxyl transferase (α and β-CT) (Menéndez and De Prado, 1999). The homomeric ACCase (common in the Poaceae family) has two identical subunits (Gornicki et al., 1997; Incledon and Hall, 1997). It has fused functional domains (NH_2_-BC-BCCCT-COOH) arranged linearly (Nikolau et al., 2003). Wheat crops lack heteromeric ACCase but have two isoforms of homomeric ACCase localised in the plastid and cytosol (Podkowinski et al., 2003; Singh et al., 2012). Both the target weed as well as the crop has homomeric ACCase still herbicides specifically target the ACCase of weed. Safeners are compounds with the ability to protect grass crops (Davies and Caseley, 1999; Hatzios and Burgos, 2004) such as wheat from herbicides by selectively enhancing the expression of enzymes responsible for herbicide metabolisms such as glutathione S-transferases (GSTs) and cytochrome P_450_ monooxygenases (P_450s_) in crops (Hatzios and Hoagland, 1989; Farago et al., 1994; Riechers et al., 2005). With the aid of safener crop plant has an intensified expression of herbicide metabolising enzymes when compared to weeds, which aids in the detoxification of crops (Riechers et al., 2005). They are usually mixed in herbicide formulations to protect crop plants from the toxic effect of herbicides. It is an unexplored phenomenon: How ACCase herbicides selectively target specific weeds in crop fields? In this study we have pondered upon the same and have analysed the sequences as well as modelled structure of *P. minor* and *T. aestivum* ACCase protein, to determine the possible difference at the herbicide binding site.

## 2 Material and methods

### 2.1 Sequence retrieval and alignment

The amino acid sequence of *P. minor* and *T. aestivum* ACCase (Q84U78 and B2ZGL3; respectively) have been retrieved from the UniProtKB/TrEMBL database (https://www.uniprot.org/uniprotkb/). A pairwise local alignment has been performed using the EMBL-EBI EMBOSS-Water tool (https://www.ebi.ac.uk/Tools/psa/emboss_water/) with the retrieved sequences, to locate the sequence identity and similarity between the two sequences.

### 2.2 Homology modelling of proteins

The sequences of *P. minor* and *T. aestivum* ACCase protein have been modelled using Modeller 10.3 (Webb and Sali, 2016), a homology modelling program. Basic Local Alignment Search Tool for protein *i.e.*, BLASTp (http://blast.ncbi.nlm.nih.gov/Blast.cgi) has been performed against the PDB database (www.rcsb.org) to identify the suitable template for modelling both the proteins. Target-template alignment has been performed and the alignment result has been utilised to generate homology models of the respective proteins. Models have been evaluated based on Discrete Optimised Protein Energy (DOPE), GA341 score and Root Mean Square Deviation (RMSD) of modelled structures with respect to their respective templates and one model has been selected for further studies. It has been followed by the refinement of selected models. Further, structures have been validated for their quality and geometrical correctness by visualising the occurrence of each residue on the Ramachandran map and SAVES server (https://saves.mbi.ucla.edu/). Packing-folding properties of protein have been evaluated *via* ProSA (Wiederstein and Sippl, 2007) web server. After validation, the modelled proteins have been energy minimised by utilizing the Schrödinger tool (Jorgensen et al., 1996; Harder et al., 2016) for their structural stability.

### 2.3 Molecular dynamics simulation

Molecular dynamic simulation of modelled native (apo) ACCase CT-domain of *P. minor* and *T. aestivum*, has been performed using Gromacs 5.1 (Hess et al., 2008) under periodic boundary conditions. The dynamic behavior of apoproteins has been explored in periodic boundary conditions with a Gromos 54A7 force field (Schmid et al., 2011, p. 7). The generated systems have been solvated with the explicit simple point charge (SPC) water (Hermans et al., 1984). In order to nullify any charge present in the system, neutralisation has been performed. Energy minimization has been performed on both systems using the steepest descent method for 50000 steps, with a convergence criterion of 100 Kcal/mol/Å. The electrostatic force has been calculated through the particle mesh Edward method (PME) (Essmann et al., 1995) and Van der Waals interactions have been calculated with a 1 nm cut-off. Equilibration of systems has been accomplished with respect to temperature as well as pressure. Temperature equilibration up to 26.85°C has been done using V-rescale temperature coupling while bar pressure has been achieved by using Parrinello-Rahman Barostat (Parrinello and Rahman, 1981). Heavy atoms present in each system have been constrained by the LINCS algorithm (Hess et al., 1997). To ensure the structural stability of proteins, a short MD simulation of 50 ns has been performed with a time step of 2 fs (Ryckaert et al., 1977). Further analysis of trajectories has been performed using various GROMACS in build scripts, and structural similarity between the two proteins has been analysed.

## 3 Results and discussions

### 3.1 Sequence analysis of proteins

**Figure 1:**
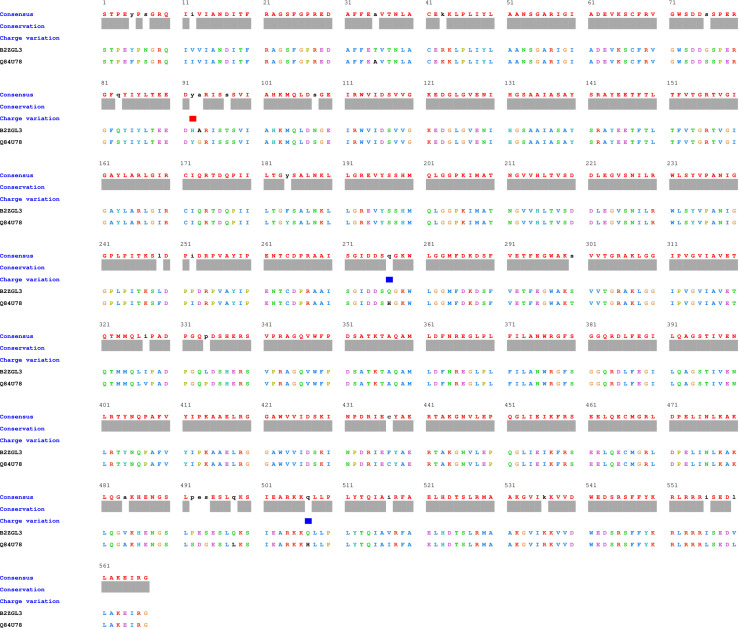
Local alignment of ACCase CT-domain of *P. minor* and *T. aestivum*.

**Figure 2:**
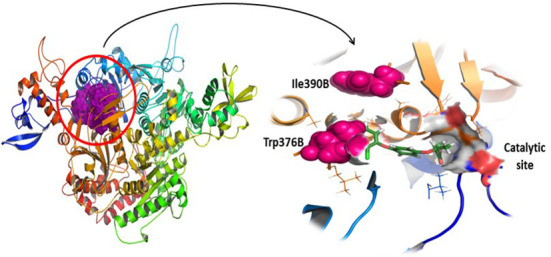
*Phalaris minor* ACCase CT-domain with special emphasis on mutations (Ile390B and Trp376B) present in the protein and a highlight on the herbicides’ catalytic site.

Protein sequences of ACCase proteins’ CT-domain of *T. aestivum* (B2ZGL3) and *P. minor* (Q84U78) have been subjected to pairwise local alignment. Pairwise sequence alignment results delineate that there are differences between the two sequences (**Figure 1**). The sequences are 93.9% identical and 97.2% similar to each other. Despite the observed similarities between the sequences, there have not been any alterations/mutations at the herbicide-binding site of proteins (**Figure 2**). The residues within a radius of 4 Å, that have an active role in herbicide binding site on both the chains of *P. minor* ACCase CT-domain are Ile130A, Ser133A, Gly159A, Ile160A, Tyr163A, Leu181A, Thr182A, Arg377B, Gly378B, Phe379B, Arg419B, Gly420B, Gly421B, Ala422B, Val224B, Val447B, Leu448B, Glu449B and Gly451B. The mutations observed at Trp376B and Ile390B in *P. minor* CT-domain sequence (as reported in *Alopecurus myosuroides* Trp2027 and Ile2041) (Raghav et al., 2016) are not at the active site but lie closer to it, thus it can be said that there is no mutation at the active site of protein around 4 Å (**Figures 1**, **2**).

### 3.2 Validation of modelled proteins

**Figure 3:**
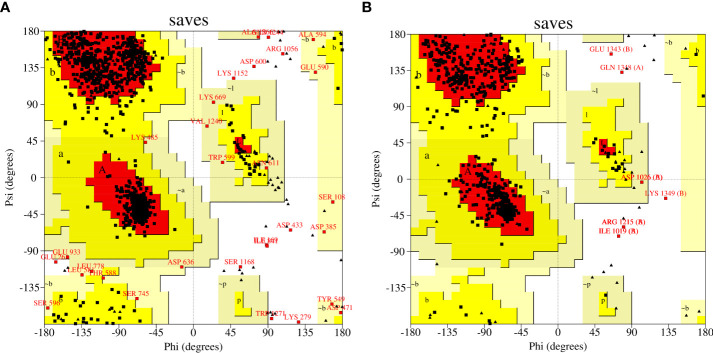
Mapping of amino acid residues’ coordinates of modelled ACCase CT-domain of **(A)** *P. minor* and **(B)** *T. aestivum* on Ramachandran plot.

**Figure 4:**
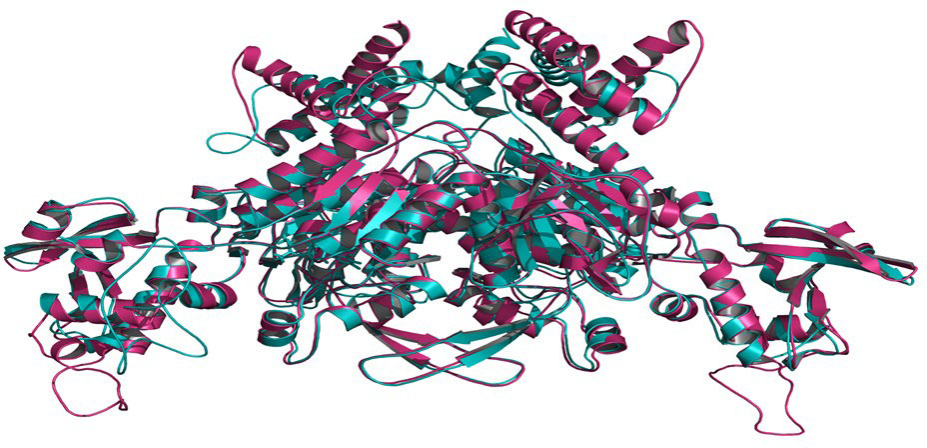
Superimposition of modelled CT-domain of ACCase protein of *P. minor* and *T. aestivum* to determine the dissimilarity between the modelled structures 0.975 Å.

Templates with PDB IDs. 1UYR (Zhang et al., 2004) and 3PGQ (Linda et al., 2010) have been chosen for modelling of ACCase CT-domain of *P. minor* and *T. aestivum*, based on the query length (92% and 99%), sequence similarity (51.42% and 45.02%) and resolution of crystal structures (2.06 Å and 2.80 Å) as mentioned in **Table 1**. Models were generated for each protein that has been later evaluated based on their DOPE, GA341 score and RMSDs, and finally, one model has been selected for further studies. Energy minimization of each modelled protein has been performed by utilising the minimization tool of the Schrodinger program (Harder et al., 2016). The modelled proteins have been energy minimised in water for 2500 steps of conjugate gradient, with parameters of the AMBER94 force field (Cornell et al., 1995). The spatial arrangement (phi-psi torsion angles) of amino acid residues of proteins has been validated by plotting them on the Ramachandran plot, which depicts that 89.5% and 90.6% of amino acids are in the highly favoured region, 9.6% and 8.8% are in the allowed region while 0.9% and 0.6% are outliers; respectively for ACCase CT-domain of *P. minor* and *T. aestivum* (**Figure 3**, **Table 2**). Protein Structure Analysis (ProSA-https://prosa.services.came.sbg.ac.at/prosa.php) web tool (to determine protein stereochemical packing of proteins) has also been utilised to validate the generated models and a ProSA Z-score of -9.96 and -9.33 has been found for the respective *P. minor* and *T. aestivum* ACCase. The modelled structure of *P. minor* and *T. aestivum* ACCase CT-domain has also been superimposed over each other (**Figure 4**) to investigate their structural similarity and an RMSD of 0.975 Å has been obtained. Besides this SAVES server (https://saves.mbi.ucla.edu/) has also been used for the validation of models, prior to a short simulation of 50ns.

**Table 1:** Selection of template for modelling of P. *minor* and *T. aestivum* ACCase CT-domain.

S. no.	CT-domain of ACCase protein	Query Protein (Uniprot ID.)	Selected template (PDB ID.)	Query coverage	Sequence identity	Template’s resolution	E-value
1.	*P. minor*	Q84U78	1UYR	92%	51.42%	2.60 Å	0.0
2.	*T. aestivum*	B2ZGL3	3PGQ	99%	45.02%	2.80 Å	8e-106

**Table 2:** Model validation for residue spatial arrangement through Ramachandran plot.

S. no.	Modelled protein	Ramachandran Plot statistics (in %)		
		Highly favoured region	Favoured Region	Disallowed Region
1.	*P. minor*	89.5	9.6%	0.9
2.	*T. aestivum*	90.6	8.8%	0.6

### 3.3 Structural similarity in ACCase CT-domain of *P. minor* and *T. Aestivum*

**Figure 5:**
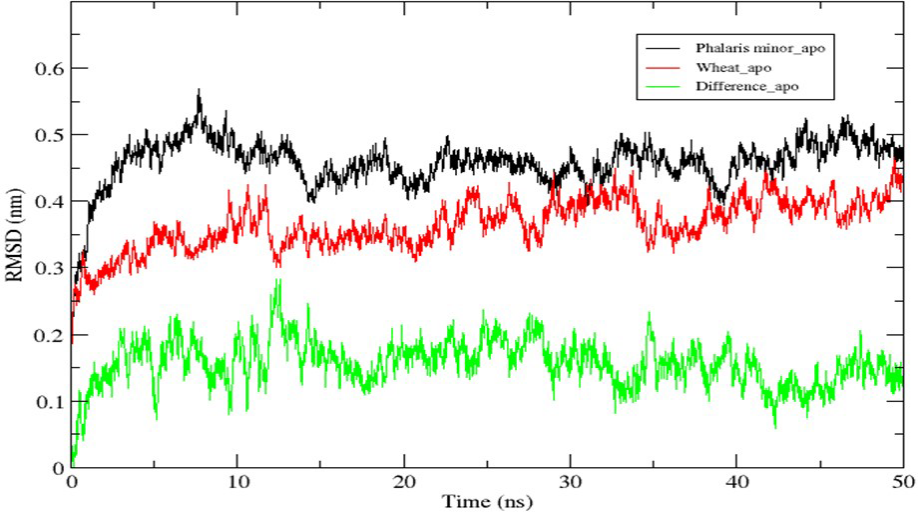
Root Mean Square Deviation (RMSD) of native ACCase CT-domain of *P. minor*, T. *aestivum* and the difference in their deviations, throughout the MD simulation of 50 ns.

The modelled proteins (*P. minor* and *T. aestivum* ACCase CT-domain) have been subjected to MD simulation, to attain the local minima state of proteins. Simulated trajectories have been subjected to various analyses, and the stability of proteins has been determined. Root Mean Square Deviations (RMSD) of proteins have been plotted to visualise the structural changes in protein throughout the simulation (**Figure 5**). No sudden shift in RMSD plots of both the proteins has been observed *i.e.*, proteins have remained stable throughout the simulation. The RMSD of proteins has been compared with each other to determine the pattern of deviation (**Figure 5**). The difference between the RMSD of proteins has an average deviation of 0.925 Å throughout the simulation timescale as shown in **Figure 4** (data plotted in green colour). Before MD simulation modelled proteins have been superimposed over each other and an RMSD of 0.975 Å has been obtained. This reveals that there is not much difference in the structure of *P. minor* and *T. aestivum* CT-domain. Herbicide-binding cavities of both proteins have been thoroughly investigated in this study and no noticeable structural differences have been identified (**Figure 1**).

Using herbicides that inhibit ACCase is one of the main strategies to selectively control grassy weeds like *P. minor*. A computational study regarding binding mode of FOP and DIM group has been conducted and no considerable variations has been observed in binding site of different weeds (Kishor and Singh, 2018; Rani et al., 2019). There are reports of resistance in various other weeds against herbicides due to mutations (Ile-2041-Val/Thr). Correlation quantification study of resistance in biotypes of *Beckmannia syzigachne* has reveille that resistance against FOP herbicides (no reversal effect on resistance due to cytochrome P450 and glutathione S-transferase *aka* GST inhibitor), cyclohexanediones (DIMs) group of herbicides and phenyl pyrazoline (DEN) herbicide is due to Ile-2041-Val resistant biotype (Wang et al., 2021). Resistance in *Lolium perenne* has also been reported due to the same mutation against pinoxaden (Ghanizadeh et al., 2022). In *Alopecurus aequalis* mutation at the same position as Thr (Ile-2041-Thr) has been reported that confers resistance to FOP and DEN (Guo et al., 2017).

In our study, all the points discussed formerly (sequence and structural similarity of proteins) converge on the fact that there is no significant difference between the two proteins’ (*P. minor* and *T. aestivum* ACCase CT-domain) sequences and structures at the binding sites. The results of the computational study, conclude that the selective behavior of herbicides against weeds is not because of sequential or structural differences in protein. However, there are reports that mutations in ACCase protein at Trp2027 and Ile2041 (Raghav et al., 2016) could result in the development of resistance in *P. minor* against prescribed herbicides. The reported mutations are not at the catalytic site of the protein but lie close to it. It has been noted that the amino acid residues present within the vicinity of 4 Å of mutation sites are part of the catalytic site, thus it could be possible that the mutation has a partial effect on the binding of herbicides which needs to be explored, as an understanding mechanism of resistance is the primary step to design strategy for weed management.

## 4 Conclusion

The mechanism by which herbicides selectively target weeds has been an intriguing research area. One of the assumptions could be that *P. minor* ACCase is different from that of *T. aestivum* ACCase which eases the herbicides in recognizing its target. However, upon analysis, it has been confirmed that there is no significant difference between the sequence of *P. minor* and *T. aestivum* CT-domain of ACCase. It has also been noted that the herbicide binding site of both proteins is conserved *i.e.*, there are no sequential or structural differences at the binding site within 4 Å of herbicide binding. The models have also been investigated concerning their structural detail, which reveals that proteins are exceedingly similar. The results rule out the assumption that herbicides recognize their target due to protein differences. Thus, a question remains unanswered about how the herbicide selectively targets the weed’s ACCase protein. The role of these structural regions has been explored through MD simulations to understand their structural integrity and effect on the binding of herbicides, still, the effect of mutation on weed resistance needs to be explored more.

## Data availability statement

The original contributions presented in the study are included in the article/supplementary material. Further inquiries can be directed to the corresponding author.

## Author contributions

The author BR and PR have equally contributed and should be considered individually and jointly as the first author. The computational work has been carried out by BR and PR. The manuscript was structured and prepared by BR and PR under the guidance of DS. All authors contributed to the article and approved the submitted version.
